# Association of FIB-4 index and clinical outcomes in critically ill patients with acute kidney injury: a cohort study

**DOI:** 10.1186/s12876-021-02071-2

**Published:** 2021-12-20

**Authors:** Yi-Yi Shi, Rui Zheng, Jie-Jie Cai, Zheng-Dong Fang, Wen-Jing Chen, Jing-Ye Pan, Song-Zan Qian

**Affiliations:** 1 Department of Anesthesiology, The First Affiliated Hospital of Wenzhou Medical University, Wenzhou, 325000 China; 2 Department of Intensive Care Unit, The First Affiliated Hospital of Wenzhou Medical University, Wenzhou, 325000 China; 3 School of the First Clinical Medical Sciences, Wenzhou Medical University, Wenzhou, 325000 China; 4 Wenzhou Key Laboratory of Critical Care and Artificial Intelligence, Wenzhou, China

**Keywords:** Acute kidney injury, Fibrosis-4 (FIB-4), Liver fibrosis, Mortality

## Abstract

**Background:**

The relationship between fibrosis-4 (FIB-4) index and clinical outcomes in patients with acute kidney injury (AKI) is unclear. We aimed to investigate the association between FIB-4 index and all-cause mortality in critically ill patients with AKI.

**Methods:**

We used data from the Multiparameter Intelligent Monitoring in Intensive Care III (MIMIC-III) database (v1.4). The FIB-4 score was calculated using the existing formulas. logistic regression model, and Cox proportional hazards model were used to assessed the relationship between the FIB-4 index and in-hospital,28-day and 90-day mortality, respectively.

**Results:**

A total of 3592 patients with AKI included in the data analysis. 395 (10.99%) patients died during hospitalization and 458 (12.74%) patients died in 28-day. During the 90-day follow-up, 893 (22.54%) patients were dead. An elevated FIB-4 value was significantly associated with increased in-hospital mortality when used as a continuous variable (odds ratio [OR] 1.183, 95% confidence interval [CI] 1.072–1.305, *P* = 0.002) and as a quartile variable (OR of Q2 to Q4 1.216–1.744, with Q1 as reference). FIB-4 was positively associated with 28-day mortality of AKI patients with hazard ratio (HR) of 1.097 (95% CI 1.008, 1.194) and 1.098 (95% 1.032, 1.167) for 90-day mortality, respectively.

**Conclusion:**

This study demonstrated the FIB-4 index is associated with clinical outcomes in critically ill patients with acute kidney injury.

**Supplementary Information:**

The online version contains supplementary material available at 10.1186/s12876-021-02071-2.

## Introduction

Acute kidney injury (AKI) is a clinical syndrome that complicates the course and worsens clinical outcomes and is a frequent occurrence among critically ill patients [1, 2]. Although substantial improvement has been achieved, no effective preventive or therapeutic options exist for AKI. Recent studies show that AKI is linked to a wide range of distant organ injuries, with the liver, heart, lungs, and intestines representing the most clinically relevant affected organs [3–5]. In most cases, AKI may be due to the development of organ interactions in the disease state. It has been hampered by a lack of clear clinical classifications for various types of AKI to find effective AKI treatments [6].

AKI patients with liver cirrhosis indicates a poor prognosis, in addition, patients with advanced fibrosis accompanied by AKI also indicate an adverse outcome. Liver fibrosis might be a sequela of hepatic or systemic inflammation [7]. Recent literature shows that non-alcoholic fatty liver disease (NAFLD) promotes the progression of chronic kidney disease (CKD), vice versa acute kidney injury (AKI) endorses the induction and progression of liver dysfunction [4]. Therefore, early attention should be paid to patients with different stages of liver fibrosis in AKI patients.

The fibrosis-4 (FIB-4) index is a good non-invasive index for evaluating the stage of liver fibrosis, and it has a good correlation. Studies have shown that FIB-4 is related to the prognosis of patients in many diseases, including heart failure, chronic obstructive pulmonary diseases (COPD), myocardial infarction, liver tumors, colorectal tumors, sepsis, even in generally people, it can also be used as a prognostic indicator[7–11]. The relationship between FIB-4 index and clinical outcomes in AKI patients is unclear. Accordingly, this study aims to investigated whether liver fibrosis assessed using FIB-4 index is associated with clinical outcomes in critically ill patients with AKI.

## Material and methods

### Data source

This retrospective cohort study was conducted using data from Multiparameter Intelligent Monitoring in Intensive Care III (MIMIC-III database v1.4). MIMIC-III is a freely available and publicly critical care database to the medical research community, which consists of about over 40,000 patients stayed in the ICU of Beth Israel Deaconess Medical Center (Boston, Massachusetts) between June 2001 and October 2012 [12]. The access to the database has been approved by the institutional review boards of both Beth Israel Deaconess Medical Center and Massachusetts Institute of Technology Affiliates. Our access to the database was approved after completion of the NIH web-based training course named ‘Protecting Human Research Participants’ (certification number: 1797305). The Institutional Review Boards of the Massachusetts Institute of Technology approved the development of this database. The Strengthening the Reporting of Observational Studies in Epidemiology statement was followed in the design of this reference study [13].

### Study population

**Fig. 1:**
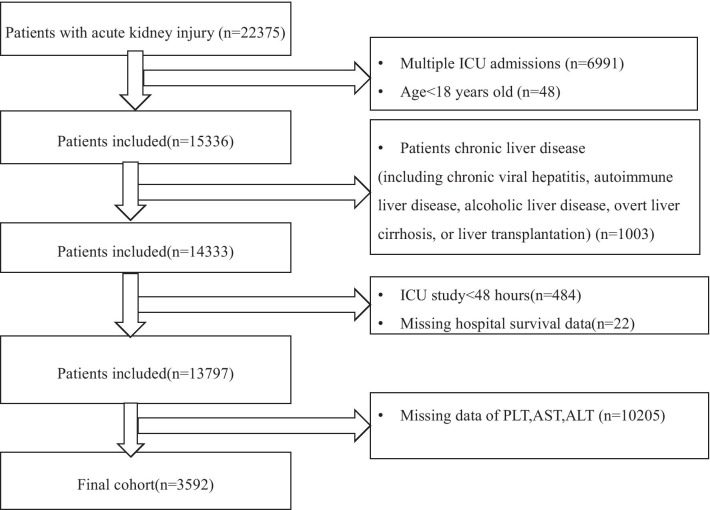
Inclusion/Exclusion criteria

The study flow diagram of the inclusion and exclusion procedure for MIMIC-III is shown in Fig. 1.The diagnosis of AKI adopts the Kidney Disease: Improving Global Outcomes (KDIGO) criteria [14]. Patients meeting the KIDGO criteria were considered eligible for study inclusion. AKI stages were defined by both serum creatinine (Scr) and the volume of urine during the first 48 h after ICU admission. The exclusion criteria were as follows: (1) age < 18 years old; (2) re-admission; (3) died within 48 h after ICU admission; (4) missing baseline aspartate aminotransferase (AST) (IU/L) or platelet count (109/L) or alanine aminotransferase (ALT)value at ICU admission, (iv) chronic liver disease (including chronic viral hepatitis, autoimmune liver disease, alcoholic liver disease, overt liver cirrhosis, or liver transplantation).

### Data collection and definitions

The data on the first day of ICU admission were extracted from MIMIC III using Structured Query Language (SQL) including age, admission ICU type, gender, ethnicity, weight, comorbidities, Simplified Acute Physiology Score II (SAPSII), Sequential Organ Failure Assessment (SOFA) score, Elixhauser comorbidity score, use of vasopressors, mechanical ventilation and renal replacement therapy (RRT), daily fluid input, fluid balance for 48 h, and certain laboratory tests and comorbidities (Table 1). All laboratory parameters that were extracted from the MIMIC-III database had been measured in the first day of ICU admission. The FIB-4 index was calculated using the following equation: FIB-4 index (Fibrosis-4 index = (Age × AST) / (Platelets × √(ALT) [15].

**Table 1:** Patient characteristics by quartiles of FIB-4 at baseline

FIB-4	Q1(0.218–1.606)	Q2(1.607–2.832)	Q3(2.833–5.373)	Q4(> 5.376)
N	991	990	990	991
**AKI stage, n (%)**				
Stage 1	442 (44.601)	432 (43.636)	393 (39.697)	358 (36.125)
Stage 2	327 (32.997)	318 (32.121)	331 (33.434)	354 (35.721)
Stage 3	222 (22.402)	240 (24.242)	266 (26.869)	279 (28.153)
Age	54.217 (18.135)	64.938 (16.388)	68.903 (15.364)	70.277 (14.536)
Gender(male), n (%)	584 (58.930)	585 (59.091)	580 (58.586)	589 (59.435)
Weight (Kg)	85.675 (26.086)	83.439 (24.102)	81.689 (24.089)	80.030 (20.430)
**Admission type, n (%)**				
MICU	517 (52.17)	478 (48.283)	429 (43.333)	386 (38.951)
CCU	152 (15.338)	184 (18.586)	208 (21.01)	205 (20.686)
SICU	144 (14.531)	133 (13.434)	121 (12.222)	115 (11.604)
Others	178 (17.962)	195 (19.697)	232 (23.434)	285 (28.759)
**Ethnicity, n (%)**				
White	652 (65.79)	690 (69.70)	698 (70.51)	670 (67.61)
Black	117 (11.81)	105 (10.61)	87 (8.79)	82 (8.27)
Asian	24 (2.42)	31 (3.13)	27 (2.73)	22 (2.22)
Others	198 (19.98)	164 (16.57)	178 (17.98)	217 (21.90)
**ICU intervention**				
Mechanical ventilation (first24hours), n (%)	274.138 (71.222)	221.546 (67.182)	187.199 (69.434)	152.168 (65.734)
Vasopressor (first24hours), n (%)	25(19–38)	37 (24–59)	58(36–101)	153(75–318)
Renal replacement therapy, n (%)	24 (17–42)	28 (17–50)	35(19–74)	63 (30–159)
Fluid input (first 48 h) (ml)	477 (48.133)	479 (48.384)	569 (57.475)	608 (61.352)
	266 (26.842)	294 (29.697)	412 (41.616)	501 (50.555)
	92 (9.284)	109 (11.01)	106 (10.707)	155 (15.641)
	1737(492–3750)	1722(531–3373)	1962 (737–3615)	2084 (856–4059)
**Fluid balance**				
Volume (first 24 h) (ml)	1239 (− 367–3484)	1214 (− 165–3692)	1744(45–4255)	2448 (407–5585)
Positive, n (%)	658 (68.973)	679 (72.311)	717 (75.793)	757 (80.361)
Volume (second 24 h) (ml)	220(− 909–2119)	280 (− 653–1822)	600(− 526–2259)	713 (− 713–2783)
Positive, n (%)	442 (54.907)	478 (58.01)	526 (62.619)	545 (63.892)
**Laboratory**				
Hemoglobin(g/L)	18.484 (5.287)	19.633 (5.046)	20.870 (5.036)	21.491 (5.545)
WBC(10^9/L)	4 (2–6)	4.5 (3–7)	6(4–8)	7(5–10)
PH	5.5 (0–12)	7(3–13)	10 (5–15)	10(5–16)
PLT(10^9/L)	11.431 (2.179)	11.078 (2.082)	10.945 (2.11)	10.693 (2.2)
AST(u/L)	12(8.55–16.4)	11.4 (8.2–15.7)	11.45 (8–16.1)	11.3(8–16.4)
ALT(u/L)	7.350 (0.104)	7.339 (0.095)	7.334 (0.103)	7.328 (0.113)
**Co-morbidities, n (%)**				
CHF	234 (23.613)	332 (33.535)	366 (36.970)	348 (35.116)
AFib	176 (17.76)	264 (26.667)	331 (33.434)	366 (36.932)
Chronic renal disease	140 (14.127)	182 (18.384)	197 (19.899)	187 (18.87)
COPD	107 (10.797)	125 (12.626)	117 (11.818)	112 (11.302)
CAD	172 (17.356)	254 (25.657)	287 (28.99)	353 (35.621)
Stoke	94 (9.485)	88 (8.889)	70 (7.071)	64 (6.458)
Malignancy	126 (12.714)	144 (14.545)	162 (16.364)	148 (14.934)
Sepsis	438 (44.198)	486 (49.091)	513 (51.818)	533 (53.784)
ARDS	52 (5.247)	54 (5.455)	58 (5.859)	59 (5.954)
**Outcomes**				
In-hospital mortality, n (%)	86 (8.678%)	128 (12.929%)	174 (17.576%)	200 (20.182%)
28-day mortality, n (%)	93 (9.384%)	144 (14.545%)	178 (17.980%)	199 (20.081%)
90-day mortality, n (%)	143 (14.430%)	207 (20.909%)	268 (27.071%)	275 (27.750%)

SAPSII score: was calculated within the first 24 h after the ICU admission using the value associated with the greatest severity of illness; SOFA score: Sequential Organ Failure Assessment; Elixhauser score: Elixhauser Comorbidity Index; WBC: white blood cell; CHF: congestive heart failure; PLT: platelet; AST: aspartate aminotransferase; ALT: alanine transaminase; AFib: atrial fibrillation; COPD: chronic obstructive pulmonary disease; CAD: coronary artery disease; ARDS: acute respiratory distress syndrome. The first values during the first day after ICU admission were recorded

Details regarding the missing values can be found in the additional file. To prevent our population selection bias, we divided the population into ALT, AST, PLT non-missing group, and missing group, then compare the baseline population characteristics between groups. We found no significant differences between the groups (Details in Additional file 1).

### Endpoints

The clinical outcomes were in-hospital,28-day, and 90-day mortality. The starting date of the follow-up was the admission date, the duration of follow-up is defined as 90 days, while the date of death was acquired from the US government's Social Security Death Index.

### Statistical analysis

The presentation of continuous variables was divided according to their distribution status, normal distribution was presented as mean ± standard deviation and skewed distribution was presented as medium (interquartile range (IQR)). Nonnormally distributed continuous variables were compared using the Wilcoxon signed-rank test. Kruskal–Wallis *H* test or Chi-squared test was employed to different FIB-4 quartile groups. Non-linear relationships between FIB-4 levels and 28-day, in-hospital, and 90-day mortality were assessed using restricted cubic spline curves respectively. The cumulative incidence of 90-day mortality was estimated using the Kaplan–Meier method according to FIB-4 quartiles. The association between FIB-4 levels with in-hospital mortality was evaluated by logistic analysis. Cox proportional hazards regression models was used to calculate the hazard ratio (HR) to estimate the association between FIB-4 levels and the risk of 90-day mortality. The regression results are reported according to FIB-4 quartiles (categorical variable) and reported by per SD increase in FIB-4 (continuous variable) using the lowest quartile 1 (Q1) as the referent to aid in interpretation.

Collinearity between variables was tested using the variance inflation factor (VIF) based on a multiple regression model [16]. As shown as in additional files, the variables with VIF > 5 were considered to exhibit collinearity. These covariates were chosen according to their relationship with the outcomes of interest or > 10% effect estimate changes [17]. We constructed four models; Model 1 adjusts for none; Model 2 adjust for: gender; weight; Admission ICU type; Model 3 adjust for: weight; SAPSII; mechanical ventilation; Vasopressor; RRT; comorbidities (including Congestive Hearts Failure (CHF), Atrial Fibrillllatation (AFib), Chronic renal disease, COPD, CAD, Stoke, malignancy, sepsis, ARDS). Model 4 adjust for: Model 3 + AKI stage. Stratified analyses by subgroup variables were presented with a fully adjusted Model 4. Log-likelihood ratio test was used to assess the interaction effects between FIB-4 and subgroup variables.

All analyses were performed with the statistical software R V.3.6.1 (R Foundation for Statistical Computing, Vienna, Austria) and EmpowerStats (http://www.empowerstats.com, X&Y Solutions, Boston, Massachusetts, USA), a two-side *p* value of < 0.05 was considered statistically significant in all analyses.

## Results

### Patient population

The study flow diagram is shown in Fig. 1. The details of missing of variables and the baseline characteristics between those with FIB-4 and without were shown in Additional file 1 STable1 and STable1. A total of 3592 patients with AKI were involved in this study according to inclusion and exclusion criteria. The median age and FIB-4 level was 67 years (IQR 53–79), 2.83 (IQR 1.61–5.38) respectively. Characteristics of the study cohort are shown in Table 1. According to FIB-4 quartile concentrations, patients with AKI were divided into four group. Patients with higher FIB-4 value were more likely to be older, male, higher SAPSII, SOFA, Elixscor score value. FIB-4 levels seemed to be positively associated with lactate, whereas it was inversely correlated with albumin, white blood cell, and PH. 

### FIB-4 and in-hospital mortality, 28-day mortality

**Fig. 2:**
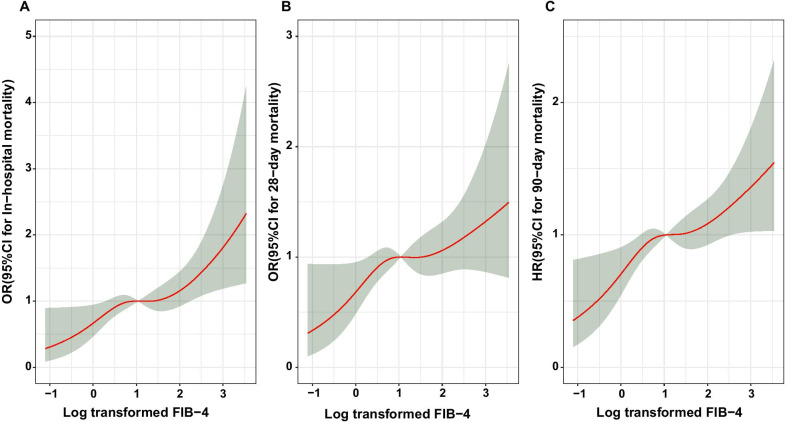
Restricted cubic spline fitting for the association between log transformed FIB-4 levels with in-hospital mortality, 28-day mortality and 90-day mortality

395 (10.99%) of the 3592 patients had a death during hospitalization. During the 28-day follow-up, 458 (12.74%) of the 3592 patients died. The results of univariate analysis and multivariate logistic regression of In-hospital mortality 28-day mortality and were shown in Additional file 1: Stable 3, Stable 4, and Stable 5, Stable 6 respectively. The restricted cubic spline curves demonstrated an S-shaped relationship between log-transformed FIB-4 and in-hospital mortality and 28-day mortality (Fig. 2A, B). We listed the effect sizes of the association between FIB-4 and in-hospital mortality and 28-day mortality in Table 2. Elevated FIB-4 value was positively associated with death risk in the unadjusted model. In Model 2, after adjusting for sociodemographic variables (gender; weight; Admission ICU type), the association between FIB-4 and in-hospital mortality and 28-day mortality was strongly significant, regardless of model III or model IV (added AKI stage and rehydration adjustment variables), the association remaining strong (Details in Table 2). In the adjusted model IV, we observed that the risk of in-hospital mortality (95% CI) among those with FIB-4 values in Q3 and Q4 were 1.443 (1.002–2.079) and 1.744 (1.215–2.505) times respectively as high as patients with FIB-4 value in Q1. The results of the adjusted model IV shown that an increased risk of 28-day mortality for patients with FIB-4 value in Q4 (OR 1.578, 95% CI 1.169–2.130) compared with the reference group. Similar results were acquired when treating FIB-4 as a continuous variable and in an adjusted model that includes age for in-hospital mortality (Details in Additional file 1: Stable 9).

**Table 2:** HRs or ORs (95% CIs) associated with FIB-4 for outcomes

	FIB-4 Q1	FIB-4Q2 P-value	FIB-4 Q3 P-value	FIB-4 Q4 P-value	1SD-increased in FIB-4 P-value
**In-hospital death**†					
Model I	1	1.563(1.171, 2.086) 0.00245	2.244 (1.704, 2.955) < 0.00001	2.661 (2.031, 3.486) < 0.00001	1.298 (1.207, 1.396) < 0.00001
Model II	1	1.456 (1.078, 1.968) 0.01437	2.128 (1.597, 2.834) < 0.00001	2.549 (1.924, 3.377) < 0.00001	1.295 (1.201, 1.396) < 0.00001
Model III	1	1.269 (0.923, 1.743) 0.14237	1.645 (1.210, 2.236) 0.00148	1.774 (1.306, 2.409) 0.00025	1.163 (1.070, 1.266) 0.00042
Model IV	1	1.216 (0.835, 1.772) 0.30829	1.443 (1.002, 2.079) 0.04906	1.744 (1.215, 2.505) 0.00258	1.183 (1.072, 1.305) 0.00081
**28-days death**†					
Model I	1	1.644 (1.246, 2.168) 0.00044	2.117 (1.619, 2.768) < 0.00001	2.426 (1.863, 3.159) < 0.00001	1.241 (1.154, 1.334) < 0.00001
Model II	1	1.583 (1.186, 2.113) 0.00182	2.056 (1.554, 2.721) < 0.00001	2.318 (1.758, 3.056) < 0.00001	1.229 (1.140, 1.325) < 0.00001
Model III	1	1.368 (1.010, 1.855) 0.04325	1.548 (1.148, 2.086) 0.00412	1.595 (1.182, 2.152) 0.00224	1.100 (1.011, 1.196) 0.02640
Model IV	1	1.362 (1.004, 1.848) 0.04672	1.532 (1.136, 2.067) 0.00519	1.578 (1.169, 2.130) 0.00286	1.097 (1.008, 1.194) 0.03214
**90-days death**‡					
Model I	1	1.511 (1.221, 1.870) 0.00015	2.016 (1.645, 2.469) < 0.00001	2.116 (1.729, 2.590) < 0.00001	1.201 (1.147, 1.257) < 0.00001
Model II	1	1.448 (1.159, 1.808) 0.00110	1.942 (1.570, 2.401) < 0.00001	2.073 (1.678, 2.560) < 0.00001	1.201 (1.145, 1.259) < 0.00001
Model III	1	1.227 (0.982, 1.534) 0.07210	1.452 (1.170, 1.801) 0.00072	1.445 (1.162, 1.798) 0.00094	1.094 (1.038, 1.154) 0.00090
Model IV	1	1.229 (0.940, 1.606) 0.13118	1.359 (1.046, 1.764) 0.02150	1.470 (1.132, 1.908) 0.00379	1.098 (1.032, 1.167) 0.00300

Adjust model I adjust for: None Sample:3962

Adjust II model adjust for: gender; weight; Admission ICU type Sample:3638

Adjust III model adjust for: weight; SAPSII score; ICU interventions in first 24 h (Mechanical ventilation; Vasopressor, RRT); Co-morbidities (AFIB; chronic renal disease; COPD; CAD; Stroke; malignancy; sepsis; ARDS) Sample:3638

Adjust IV model adjust for: Adjust III + AKI stages. Sample:2436

^†^Values are ORs (95% CIs) ‡ Values are HRs (95% CIs)

**Fig. 3:**
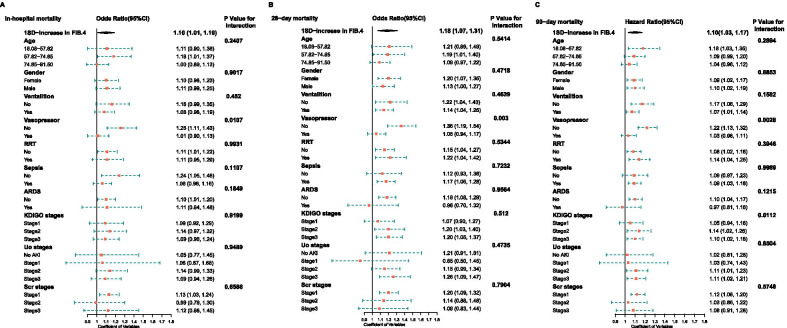
Stratified analysis of FIB-4 value after and in-hospital mortality, 28-day mortality and 90-day mortality based on model IV. Model IV was adjusted for weight, SAPSII score, some ICU interventions in first 24 h (Mechanical ventilation, Vasopressor,Renal replacement therapy),some Co-morbidities(AFIB, Chronic renal disease, COPD, CAD, Stroke, malignancy, sepsis, ARDS) and AKI stages a (None of the stratified variables) RRT: Renal replacement therapy, ARDS: acute respiratory distress syndrome, KDIGO: Kidney Disease: Improving Global Outcomes, UO: Urine output, Scr: creatinine

Subgroup analyses was performed to evaluate the robustness of the association between FIB-4 levels and in-hospital mortality and 28-day mortality respectively. In the adjusted model IV, the results of stratified analysis showed that the association of FIB-4 value was similar in the most of the sub-populations (Fig. 3A, B). Regardless of whether the outcome variable is in-hospital or 28-day mortality, and even in 90-day mortality, the Effect sizes of patients who used vasopressor in first 24 h were less than patients who have not used vasopressor (P value for interaction < 0.05).

### FIB-4 and 90-day mortality

**Fig. 4:**
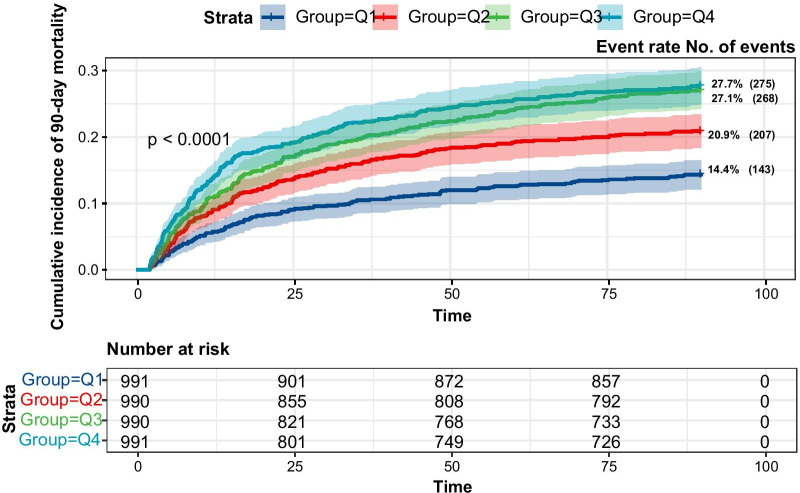
Kaplan–Meier plot of 90-day mortality across FIB-4 quartiles

During the 90-day follow-up, 893 (22.54%) patients died. The results of univariate analysis and multivariate COX regression of 90-day mortality were shown in Additional file 1: Stable 7 and Stable 8. The result of restricted cubic spline curves also showed an S-shaped relationship with 90-day mortality (Fig. 2C). The cumulative incidences of mortality at 90 days were 14.4%, 20.9%, 27.1%, and 27.7% for patients with FIB-4 values in Q1, Q2, Q3, and Q4, respectively (Fig. 4). Elevated FIB-4 was strongly associated with 90-day mortality in the unadjusted model I (Table 2). Compared with the reference group we also observed the increased risk of death for patients with FIB-4 values in Q4 (HR 1.445; 95% CI 1.162–1.798) in the result of adjusted model III. The HR was slightly attenuated (HR 1.470, 95% CI 1.13–1.908) after adjusting the AKI stage in adjusted model IV. When analyzing FIB-4 as a continuous variable, the association remained with a 9.8% increased risk of death for each SD increase in FIB-4 (95% CI 1.032–1.167). As shown in STable9, after adjusting the age, the HR was slightly attenuated (HR 1.068, 95% CI 1.010–1.129).

## Discussion

To our knowledge, we first reported that liver fibrosis scoring systems FIB-4 index is associated with all-cause mortality in critically ill patients with AKI. In general, the results showed that individuals with higher FIB-4 value suffered from an increased risk of mortality, which showed a strong and positive association with the 28-day mortality, 90-day mortality and in-hospital mortality after adjusting for other covariates. The restricted cubic spline curves demonstrated an S-shaped relationship between log-transformed FIB-4 and clinical outcomes. Additionally, the adverse association of FIB-4 index with mortality was consistent in subgroups defined according to age, gender, ventilation, RRT, sepsis, ARDS, AKI stages.

AKI is a heterogeneous clinical syndrome, mainly manifested as an increase in serum creatinine, a sudden decrease in glomerular filtration rate, and a decrease in urine output [2]. Studies have shown that there is an important interaction between kidney and heart, kidney and lung, kidney and liver [3, 18, 19]. The liver function is often compromised in patients with AKI [19]. Studies have confirmed that patients with liver cirrhosis are prone to acute kidney injury and further progress to hepatorenal syndrome with high mortality [20]. Distant organ liver damage after AKI remains a serious clinical setting with high mortality [21]. Knowing the characteristics of this kidney-liver crosstalk is crucial for handling the complications induced by this vicious circle [5].

FIB-4 is a simple and easy to obtain index, the parameters involved age, AST, ALT, and platelets. It has potential strengths for clinical application. A recent study investigating the prognosis value of FIB-4 in sepsis showed that an elevated FIB 4 index is associated with adverse clinical outcomes [11]. Moreover, circulating endotoxin an pro-inflammatory cytokines impair liver function in sepsis, further producing synergistic negative effects on patients with cirrhosis [22]. In patients with COVID-19 showed that the high FIB-4 group had a higher need of mechanical ventilation and higher in-hospital mortality, FIB-4 ≥ 2.67 was associated with increased 30-day mortality (OR 8.4; 95% CI 2.23–31.7) [23]. Ischemia–reperfusion (IR) is a common risk factor that causes AKI, a study by Shang et al. suggested that renal IR can directly activate nuclear transcription factor kappa B (NF-κB) and induce acute production of pro-inflammatory cytokines in the liver, Renal IR-induced hepatic inflammatory response may contribute to impaired systemic inflammation and liver function [19]. Kidney and liver crosstalk contributes to metabolic homeostasis and affects the inflammatory response in liver, related pathways and fibrotic changes [24]. The association between FIB-4 index and AKI patients in our study shown that there may be a certain correlation between liver and kidney damage. Higher FIB-4 indicates an advance liver fibrosis, which is related to chronic inflammation. For patients with AKI, the interaction between kidney and liver may aggravate the deterioration of liver function, which may lead to multiple organ failure. Our findings also found that as the Fib-4 value increased, the use of mechanical ventilator, the use of vasoactive drugs, and the need for RRT increased significantly. In addition, we also studied the stratified analysis based on KIDGO staging standard. In the adjusted model IV, the result of stratified analysis showed that the association of FIB-4 levels was similar across most of the sub-populations. Greater knowledge of the physiologic relationship between kidney and liver may open avenues for specific therapies of AKI [25].

Our study has several strengths. Firstly, our sample size was large, we divided the patients into more groups on the basis of the FIB-4 value and to investigate the association of FIB-4 value with all-cause death. Secondly, we used a Cox regression model with a restricted cubic spline to assessed the relationship between the baseline FIB-4 value (as a continuous variable) and the risk of all-cause mortality. A S-shaped relationship between FIB-4 value and clinical outcomes were consistent with the results of the multivariate Cox regression analysis.

There were several important limitations in our study. First of all, this was a single-center retrospective study, there were some data missing and selection bias. However, we set strict inclusion standards to control those possible bias. Secondly, Fib-4 is obtained by simple calculation, although it had a strong correlation with liver fibrosis stages, it may overestimate or underestimate the degree of liver fibrosis, which will affect the conclusion. It is necessary to further study the relationship between the basic liver condition and the prognosis of AKI population. Thirdly, the outcome of our study was the all-cause mortality, we did not analyze the cause of death, and did not explore the impact of the underlying disease on the outcome. Fourthly, Actually, the OR or HR calculated in this study is quite small (only about 1.1–1.2), although the analyses were statistically significant, probably due to the large sample size. Even we used quartile for analyses, the ORs for each quartile variable were only 1.2–1.7 (OR of Q2 to Q4: 1.216–1.744, with Q1 as reference). Therefore, further prospective studies should be warranted to validate our findings by determining fibrosis stage using “gold” standard.

## Conclusions

AKI patients with a higher degree of liver fibrosis have a higher in-hospital mortality and 90-day mortality. FIB-4 is a simple index that can be obtained daily, which is associated with clinical outcomes in critically ill patients with acute kidney injury.

## Supplementary Information

**Additional file 1**.** STable 1**. Missing of variables.**STable 2**. Baseline characteristics between those with FIB-4 and without.**STable 3**. Univariate analysis of In-hospital mortality.**STable 4**. Multivariate logistic regression of in-hospital mortality.**STable 5**. Univariate analysis of 28-day mortality.**STable 6**. Multivariate logistic regression of 28-day mortality.**STable 7**. Univariate analysis of 90-day mortality.**STable 8**. Multivariate COX regression of 90-day mortality.**STable 9**. The associated with FIB-4 for outcomes adjusted age

## Data Availability

The datasets used and/or analyzed during the current study are available from the corresponding author on reasonable request.

## Abbreviations

FIB-4: Fibrosis-4
MIMIC-III: Multiparameter Intelligent Monitoring in Intensive Care III
AKI: Acute kidney injury
OR: Odds ratio
CI: Confidence interval
HR: Hazard ratio
NAFLD: Non-alcoholic fatty liver disease
CKD: Chronic kidney disease
COPD: Chronic obstructive pulmonary diseases
KDIGO: Kidney Disease: Improving Global Outcomes
AST: Aspartate aminotransferase
ALT: Alanine aminotransferase
SQL: Structured Query Language
SOFA: Sequential Organ Failure Assessment
SAPSII: Simplified Acute Physiology Score II
IQR: Interquartile range
RRT: Renal replacement therapy
CHF: Congestive Hearts Failure
AFib: Atrial Fibrillllatation
ARDS: Acute respiratory distress syndrome
NF-κB: Nuclear transcription factor kappa B
IR: Ischemia–reperfusion
