# Supplementary material for: Association of FIB-4 index and clinical outcomes in critically ill patients with acute kidney injury: a cohort study
Source: BMC Gastroenterol. 2021 Dec 20;21:483. doi: 10.1186/s12876-021-02071-2 (PMC8685308; doi:10.1186/s12876-021-02071-2)
Supplement: Supplementary file 1 — Additional file 1. STable 1. Missing of variables.STable 2. Baseline characteristics between those with FIB-4 and without.STable 3. Univariate analysis of In-hospital mortality.STable 4. Multivariate logistic regression of in-hospital mortality.STable 5. Univariate analysis of 28-day mortality.STable 6. Multivariate logistic regression of 28-day mortality.STable 7. Univariate analysis of 90-day mortality.STable 8. Multivariate COX regression of 90-day mortality.STable 9. The associated with FIB-4 for outcomes adjusted age [file 12876_2021_2071_MOESM1_ESM.docx]

Supplementary material

STable1 Missing of variables

STable2 Baseline characteristics between those with FIB-4 and without

Stable3 Univariate analysis of In-hospital mortality

Stable4 Multivariate logistic regression of in-hospital mortality

Stable5 Univariate analysis of 28-day mortality

Stable6 Multivariate logistic regression of 28-day mortality

Stable7 Univariate analysis of 90-day mortality

Stable8 Multivariate COX regression of 90-day mortality

Stable9: The associated with FIB-4 for outcomes adjusted age

STable1 Missing of variables

| Variables | Not Missing | Missing |
| --- | --- | --- |
| ID | 3962 | 0 |
| In-hospital mortality | 3962 | 0 |
| 28-day mortality | 3962 | 0 |
| 90-day mortality | 3962 | 0 |
| 90-day mortality time | 3962 | 0 |
| Admission type | 3962 | 0 |
| age | 3962 | 0 |
| gender | 3962 | 0 |
| weight | 3638 | 324 |
| SAPSII score | 3962 | 0 |
| SOFA score | 3962 | 0 |
| Elixhauser score | 3959 | 3 |
| Mechanical ventilation | 3962 | 0 |
| Vasopressor | 3962 | 0 |
| Renal replacement therapy | 3962 | 0 |
| CHF | 3962 | 0 |
| AFIB | 3962 | 0 |
| Chronic renal disease | 3962 | 0 |
| COPD | 3962 | 0 |
| CAD | 3962 | 0 |
| Stoke | 3962 | 0 |
| Malignancy | 3962 | 0 |
| Sepsis | 3962 | 0 |
| ARDS | 3962 | 0 |
| AKI stage | 3962 | 0 |
| Fluid input (first 48hours) | 3633 | 329 |
| Data on fluid balance (first 24hours) | 3781 | 181 |
| Data on fluid balance (second 24hours) | 3322 | 640 |
| hemoglobin | 3952 | 10 |
| Platelet | 3962 | 0 |
| AST | 3962 | 0 |
| ALT | 3962 | 0 |
| WBC | 3958 | 4 |

STable2 Baseline characteristics between those with FIB-4 and without

| Variables | Not Missing FIB-4 | Missing FIB-4 | P-value |
| --- | --- | --- | --- |
| N | 3592 | 10205 |  |
| Age | 65.785 ± 17.370 | 65.716 ± 17.169 | 0.836 |
| Weight | 81.392 ± 24.950 | 81.905 ± 23.378 | 0.289 |
| SAPSII score | 19.516 ± 5.014 | 19.551 ± 5.140 | 0.721 |
| SOFA score | 4.909 ± 3.016 | 4.966 ± 3.083 | 0.335 |
| Elixhauser score | 8.043 ± 7.882 | 7.868 ± 7.611 | 0.242 |
| Hemoglobin | 10.668 ± 2.127 | 10.688 ± 2.311 | 0.648 |
| WBC | 12.710 ± 7.945 | 12.803 ± 10.272 | 0.628 |
| PH | 7.358 ± 0.096 | 7.357 ± 0.098 | 0.478 |
| Fluid input  (first 48hours) | 2139.256 ± 6367.434 | 2023.101 ± 2284.867 | 0.139 |
| Fluid balance (first 24hours) | 1905.378 ± 3438.862 | 1860.760 ± 3367.458 | 0.508 |
| Fluid balance (second 24hours) | 669.596 ± 6615.852 | 532.951 ± 2483.373 | 0.115 |
| Admission type, n (%) |  |  | 0.167 |
| MICU | 1343 (37.368%) | 3837 (37.607%) |  |
| CCU | 517 (14.385%) | 1590 (15.584%) |  |
| SICU | 451 (12.549%) | 1311 (12.849%) |  |
| others | 1283 (35.698%) | 3465 (33.961%) |  |
| Gender |  |  | 0.019 |
| Female | 1443 (40.150%) | 4325 (42.389%) |  |
| Male | 2151 (59.850%) | 5878 (57.611%) |  |
| Mechanical ventilation | 1910 (53.144%) | 5357 (52.504%) | 0.509 |
| Vasopressor | 1327 (36.923%) | 3753 (36.783%) | 0.882 |
| RRT | 346 (9.627%) | 1003 (9.830%) | 0.724 |
| CHF | 1129 (31.413%) | 3187 (31.236%) | 0.843 |
| AFIB | 1095 (30.467%) | 3074 (30.128%) | 0.703 |
| Chronic renal disease | 616 (17.140%) | 1734 (16.995%) | 0.843 |
| COPD | 465 (12.938%) | 1251 (12.261%) | 0.29 |
| CAD | 1184 (32.944%) | 3242 (31.775%) | 0.197 |
| Stroke | 320 (8.904%) | 780 (7.645%) | 0.017 |
| Malignancy | 550 (15.303%) | 1612 (15.799%) | 0.482 |
| Sepsis | 1335 (37.145%) | 3859 (37.822%) | 0.471 |
| ARDS | 114 (3.172%) | 351 (3.440%) | 0.444 |
| 28-day mortality | 458 (12.743%) | 1296 (12.702%) | 0.949 |
| 90-day mortality | 665 (18.503%) | 1880 (18.426%) | 0.918 |
| In-hospital mortality | 395 (10.991%) | 1099 (10.771%) | 0.716 |
| AKI stage |  |  | 0.264 |
| Stage 1 | 1515 (42.154%) | 4288 (42.027%) |  |
| Stage 2 | 1181 (32.860%) | 3238 (31.736%) |  |
| Stage 3 | 898 (24.986%) | 2677 (26.237%) |  |

Abbreviations

SAPSII score: was calculated within the first 24 h after the ICU admission using the value associated with the greatest severity of illness；SOFA score: Sequential Organ Failure Assessment；Elixhauser score: Elixhauser Comorbidity Index；WBC: white blood cell；CHF: congestive heart failure；PLT: platelet；AST: aspartate aminotransferase；ALT: alanine transaminase；AFIB: atrial fibrillation；COPD: chronic obstructive pulmonary disease；CAD: coronary artery disease；ARDS: acute respiratory distress syndrome.；The first values during the first day after ICU admission were recorded

Stable3 Univariate analysis of In-hospital mortality

| Covariates | exp(beta) | 95%CI Low | 95%CI Upp | P. value |
| --- | --- | --- | --- | --- |
| Age | 1.0276 | 1.0217 | 1.0336 | <0.0001 |
| Gender | 1.0168 | 0.8508 | 1.2153 | 0.8545 |
| Weight | 0.9943 | 0.9902 | 0.9984 | 0.0064 |
| SAPSII score | 1.1479 | 1.1282 | 1.1681 | <0.0001 |
| SOFA score | 1.1988 | 1.1689 | 1.2294 | <0.0001 |
| Elixhauser score | 1.0779 | 1.0666 | 1.0894 | <0.0001 |
| Mechanical ventilation | 2.4012 | 1.9839 | 2.9063 | <0.0001 |
| Vasopressor | 2.2261 | 1.8650 | 2.6572 | <0.0001 |
| Renal replacement therapy | 2.4659 | 1.9653 | 3.0941 | <0.0001 |
| CHF | 1.7696 | 1.4799 | 2.1160 | <0.0001 |
| AFIB | 1.9044 | 1.5886 | 2.2830 | <0.0001 |
| Chronic renal disease | 1.1302 | 0.9044 | 1.4124 | 0.2817 |
| CODP | 1.5105 | 1.1795 | 1.9344 | 0.0011 |
| CAD | 0.7820 | 0.6361 | 0.9614 | 0.0196 |
| Stroke | 2.2156 | 1.6955 | 2.8954 | <0.0001 |
| Malignancy | 1.8906 | 1.5207 | 2.3506 | <0.0001 |
| Sepsis | 2.5350 | 2.1027 | 3.0561 | <0.0001 |
| ARDS | 1.7680 | 1.2767 | 2.4485 | 0.0006 |
| AKI stage |  |  |  |  |
| Stage2 | 1.6647 | 1.3323 | 2.0799 | <0.0001 |
| Stage3 | 2.7678 | 2.2196 | 3.4514 | <0.0001 |
| Fluid input(first 48hours) | 1.0000 | 1.0000 | 1.0001 | 0.0626 |
| Fluid balance (first 24 hours) | 1.0001 | 1.0000 | 1.0001 | <0.0001 |
| Hemoglobin | 0.9689 | 0.9301 | 1.0092 | 0.1289 |
| WBC | 1.0252 | 1.0158 | 1.0347 | <0.0001 |
| PH | 0.1510 | 0.0620 | 0.3681 | <0.0001 |

Stable4 Multivariate logistic regression of in-hospital mortality

| Exposure | In-hospital mortality OR(95%CI） |
| --- | --- |
| FIB.4.zcore | 1.183 (1.072, 1.305) 0.00081 |
| Weight | 0.989 (0.984, 0.994) 0.00007 |
| SAPSII score | 1.077 (1.048, 1.106) <0.00001 |
| Mechanical ventilation | 1.312 (0.967, 1.779) 0.08102 |
| Vasopressor | 1.113 (0.857, 1.446) 0.42280 |
| Renal replacement therapy | 1.535 (1.078, 2.184) 0.01733 |
| CHF | 1.407 (1.089, 1.818) 0.00899 |
| AFIB | 1.370 (1.066, 1.760) 0.01395 |
| Chronic renal disease | 0.718 (0.514, 1.005) 0.05316 |
| COPD | 1.325 (0.946, 1.854) 0.10119 |
| CAD | 0.513 (0.379, 0.694) 0.00002 |
| Stroke | 2.456 (1.713, 3.521) <0.00001 |
| Malignancy | 1.811 (1.333, 2.460) 0.00015 |
| sepsis | 1.803 (1.391, 2.338) <0.00001 |
| ARDS | 1.224 (0.804, 1.865) 0.34608 |
| AKI stage |  |
| 1 | 1.0 |
| 2 | 0.606 (0.293, 1.254) 0.17689 |
| 3 | 0.852 (0.311, 2.333) 0.75529 |

Stable5 Univariate analysis of 28-day mortality

| Covariates | exp(beta) | 95%CI Low | 95%CI Upp | P.value |
| --- | --- | --- | --- | --- |
| Age | 1.0326 | 1.0266 | 1.0387 | <0.0001 |
| Gender | 1.0719 | 0.8991 | 1.2779 | 0.4387 |
| Weight | 0.9931 | 0.9891 | 0.9972 | 0.0009 |
| SAPSII score | 1.1405 | 1.1213 | 1.1600 | <0.0001 |
| SOFA score | 1.1769 | 1.1483 | 1.2063 | <0.0001 |
| Elixhauser score | 1.0809 | 1.0696 | 1.0923 | <0.0001 |
| Mechanical ventilation | 1.8955 | 1.5815 | 2.2718 | <0.0001 |
| Vasopressor | 2.0081 | 1.6881 | 2.3887 | <0.0001 |
| Renal replacement therapy | 1.9111 | 1.5141 | 2.4121 | <0.0001 |
| CHF | 1.8761 | 1.5742 | 2.2359 | <0.0001 |
| AFIB | 1.9214 | 1.6077 | 2.2964 | <0.0001 |
| Chronic renal disease | 1.2027 | 0.9687 | 1.4932 | 0.0945 |
| CODP | 1.8636 | 1.4747 | 2.3550 | <0.0001 |
| CAD | 0.9034 | 0.7416 | 1.1004 | 0.3127 |
| Stroke | 2.1287 | 1.6315 | 2.7775 | <0.0001 |
| Malignancy | 2.0946 | 1.6955 | 2.5877 | <0.0001 |
| Sepsis | 2.1346 | 1.7839 | 2.5543 | <0.0001 |
| ARDS | 1.6270 | 1.1730 | 2.2568 | 0.0035 |
| AKI stage |  |  |  |  |
| Stage2 | 1.5636 | 1.2604 | 1.9396 | <0.0001 |
| Stage3 | 2.4685 | 1.9902 | 3.0616 | <0.0001 |
| Fluid input (first 48hours) | 1.0000 | 1.0000 | 1.0000 | 0.1622 |
| Fluid balance (first 24 hours) | 1.0001 | 1.0000 | 1.0001 | <0.0001 |
| Hemoglobin | 0.9479 | 0.9105 | 0.9869 | 0.0093 |
| WBC | 1.0264 | 1.0171 | 1.0359 | <0.0001 |
| PH | 0.1538 | 0.0632 | 0.3742 | <0.0001 |

Stable6 Multivariate logistic regression of 28-day mortality

| Exposure | OR(95%CI）P-Value |
| --- | --- |
| FIB.4. score | 1.097 (1.008, 1.194) 0.03214 |
| Weight | 0.992 (0.988, 0.996) 0.00036 |
| SAPSII score | 1.102 (1.077, 1.127) <0.00001 |
| Mechanical ventilation | 0.961 (0.755, 1.223) 0.74720 |
| Vasopressor | 1.269 (1.023, 1.575) 0.03062 |
| Renal replacement therapy | 1.247 (0.934, 1.666) 0.13434 |
| CHF | 1.528 (1.239, 1.885) 0.00007 |
| AFIB | 1.354 (1.099, 1.667) 0.00440 |
| Chronic renal disease | 0.842 (0.650, 1.090) 0.19138 |
| COPD | 1.696 (1.298, 2.216) 0.00011 |
| CAD | 0.756 (0.598, 0.955) 0.01894 |
| Stroke | 2.765 (2.037, 3.753) <0.00001 |
| Malignancy | 2.234 (1.754, 2.846) <0.00001 |
| sepsis | 1.430 (1.163, 1.757) 0.00068 |
| ARDS | 1.342 (0.925, 1.947) 0.12184 |
| AKI stage |  |
| 1 | 1.0 |
| 2 | 1.294 (1.016, 1.649) 0.03676 |
| 3 | 1.565 (1.210, 2.024) 0.00065 |

Stable7 Univariate analysis of 90-day mortality

| Covariates | exp(beta) | 95%CI Low | 95%CI Upp | P.value |
| --- | --- | --- | --- | --- |
| Age | 1.0317 | 1.0271 | 1.0363 | <0.0001 |
| Gender | 1.0437 | 0.9129 | 1.1931 | 0.5312 |
| Weight | 0.9926 | 0.9894 | 0.9958 | <0.0001 |
| SAPSII score | 1.1168 | 1.1038 | 1.1300 | <0.0001 |
| SOFA score | 1.1521 | 1.1323 | 1.1722 | <0.0001 |
| Elixhauser score | 1.0703 | 1.0628 | 1.0779 | <0.0001 |
| Mechanical ventilation | 1.5733 | 1.3734 | 1.8023 | <0.0001 |
| Vasopressor | 1.7778 | 1.5591 | 2.0270 | <0.0001 |
| Renal replacement therapy | 1.9310 | 1.6338 | 2.2823 | <0.0001 |
| CHF | 1.8124 | 1.5884 | 2.0680 | <0.0001 |
| AFIB | 1.7692 | 1.5473 | 2.0229 | <0.0001 |
| Chronic renal disease | 1.3462 | 1.1496 | 1.5764 | 0.0002 |
| CODP | 1.5398 | 1.2869 | 1.8423 | <0.0001 |
| CAD | 0.9065 | 0.7796 | 1.0541 | 0.2023 |
| Stroke | 1.7434 | 1.4245 | 2.1336 | <0.0001 |
| Malignancy | 2.3077 | 1.9881 | 2.6786 | <0.0001 |
| Sepsis | 2.0792 | 1.8107 | 2.3875 | <0.0001 |
| ARDS | 1.4647 | 1.1438 | 1.8756 | 0.0025 |
| AKI stage |  |  |  |  |
| Stage2 | 1.3293 | 1.1296 | 1.5644 | 0.0006 |
| Stage3 | 1.9689 | 1.6764 | 2.3124 | <0.0001 |
| Fluid input (first 48hours) | 1.0000 | 1.0000 | 1.0000 | 0.0605 |
| Fluid balance (first 24 hours) | 1.0000 | 1.0000 | 1.0001 | <0.0001 |
| Hemoglobin | 0.9354 | 0.9068 | 0.9648 | <0.0001 |
| WBC | 1.0196 | 1.0141 | 1.0251 | <0.0001 |
| PH | 0.3559 | 0.1782 | 0.7107 | 0.0034 |

Stable8 Multivariate COX regression of 90-day mortality

| Exposure | HR (95%CI) P-value |
| --- | --- |
| FIB.4. score | 1.098 (1.032, 1.167) 0.00300 |
| Weight | 0.989 (0.985, 0.993) <0.00001 |
| SAPSII score | 1.069 (1.050, 1.089) <0.00001 |
| Mechanical ventilation | 0.937 (0.760, 1.155) 0.53993 |
| Vasopressor | 1.085 (0.900, 1.308) 0.39320 |
| Renal replacement therapy | 1.133 (0.884, 1.451) 0.32393 |
| CHF | 1.487 (1.246, 1.774) 0.00001 |
| AFIB | 1.258 (1.056, 1.497) 0.01000 |
| Chronic renal disease | 0.917 (0.736, 1.143) 0.44107 |
| COPD | 1.372 (1.094, 1.722) 0.00624 |
| CAD | 0.653 (0.530, 0.804) 0.00006 |
| Stroke | 1.837 (1.435, 2.351) <0.00001 |
| Malignancy | 2.021 (1.661, 2.460) <0.00001 |
| sepsis | 1.484 (1.231, 1.789) 0.00004 |
| ARDS | 1.201 (0.896, 1.611) 0.22015 |
| AKI stage |  |
| 1 | 1.0 |
| 2 | 0.930 (0.566, 1.528) 0.77581 |
| 3 | 1.110 (0.575, 2.143) 0.75535 |

| Stable9: The associated with FIB-4 for outcomes adjusted age. | | |
| --- | --- | --- |
|  | Univariable※ | Multivariable※ |
| In-hospital death† |  |  |
| FIB-4 | 1.163 (1.070, 1.266) 0.00042 | 1.134 (1.041, 1.234) 0.00377 |
| age | 1.018 (1.010, 1.025) <0.00001 | 1.016 (1.008, 1.024) 0.00005 |
| 28-days death† |  |  |
| FIB-4 | 1.100 (1.011, 1.196) 0.02640 | 1.069 (0.981, 1.164) 0.12762 |
| age | 1.019 (1.011, 1.027) <0.00001 | 1.018 (1.010, 1.026) <0.00001 |
| 90-days death‡ |  |  |
| FIB-4 | 1.094 (1.038, 1.154) 0.00090 | 1.068 (1.010, 1.129) 0.02111 |
| age | 1.020 (1.015, 1.026) <0.00001 | 1.020 (1.014, 1.026) <0.00001 |
| adjust for: weight; SAPSII score; ICU interventions in first 24hours (Mechanical ventilation; Vasopressor, RRT); Co-morbidities (AFIB; chronic renal disease; COPD; CAD; Stroke; malignancy; sepsis; ARDS)  †Values are ORs (95% CIs) ‡ Values are HRs (95% CIs)  ※1SD-increased in FIB-4 P-value | | |
